# msiDBN: A Method of Identifying Critical Proteins in Dynamic PPI Networks

**DOI:** 10.1155/2014/138410

**Published:** 2014-04-02

**Authors:** Yuan Zhang, Nan Du, Kang Li, Jinchao Feng, Kebin Jia, Aidong Zhang

**Affiliations:** ^1^College of Electronic Information and Control Engineering, Beijing University of Technology, Beijing 100124, China; ^2^Department of Computer Science and Engineering, State University of New York at Buffalo, Buffalo, NY 14260, USA

## Abstract

Dynamics of protein-protein interactions (PPIs) reveals the recondite principles of biological processes inside a cell. Shown in a wealth of study, just a small group of proteins, rather than the majority, play more essential roles at crucial points of biological processes. This present work focuses on identifying these critical proteins exhibiting dramatic structural changes in dynamic PPI networks. First, a comprehensive way of modeling the dynamic PPIs is presented which simultaneously analyzes the activity of proteins and assembles the dynamic coregulation correlation between proteins at each time point. Second, a novel method is proposed, named msiDBN, which models a common representation of multiple PPI networks using a deep belief network framework and analyzes the reconstruction errors and the variabilities across the time courses in the biological process. Experiments were implemented on data of yeast cell cycles. We evaluated our network construction method by comparing the functional representations of the derived networks with two other traditional construction methods. The ranking results of critical proteins in msiDBN were compared with the results from the baseline methods. The results of comparison showed that msiDBN had better reconstruction rate and identified more proteins of critical value to yeast cell cycle process.

## 1. Introduction

A biological process is a complexity of spatial and temporal interactions among innumerable molecules. Understanding dynamic biological processes and revealing the mechanisms behind dynamic systems are of great value for a wide variety of important biological and medical issues, such as understanding aging, cancers, and other perplexing diseases. Dynamic biological network mining has attracted increasing attention from biologists in the past few years [1–3]. For example, we want to know which gene or protein is of critical effect to disease development. In this domain, microarray gene expression data offers useful dynamic information and is generally exploited to locate differentially expressed genes that may be related to specific abnormal conditions. A few tools are available for finding differentially expressed genes under varying conditions among which statistical methods are widely accepted, including methods based on *t*-test and SAM [4, 5]. However, change level of gene expression is such a representation that is far from satisfaction to explain the complex dynamic mechanism, considering that it is not capable of investigating the dynamic changes of relationships of proteins in consecutive protein-protein interaction networks (PPINs). For instance, methods based on sole gene expression analysis cannot capture genes with medium expression, but in contrast more accurate and complete understanding can be achieved by putting them into the PPIN.

There are mainly two challenges in dynamic network analysis. The first one is to construct the dynamic networks that accurately model the dynamic processes. Proteins perform their functions at specific times under distinguished conditions, which we call them in their active forms. Dynamic PPINs reveal the instant relationships of functional proteins. However, the publicly available PPI datasets are mostly aggregates of all possible interactions obtained under different examined conditions or time points [6] and are oblivious to the temporal changes of these networks. Dynamic analysis involves extracting dynamic PPI networks from these known datasets and the methods mainly fall into two directions. The first way is based on the differential coexpression correlations. Studies [7] have shown that highly positive coexpressed proteins tend to form the most static modules appearing at all times and at the center of which there are some hubs with high degree being referred to as “party” hubs. Further, some less positive coexpressed proteins interact at particular time points, the hubs therefore being referred to as “date” hubs that are believed to cause dynamic interactions and plausibly induce aberrant pathways and molecular disorders. Taylor et al. [8] also observed multimodal distribution of correlation coefficients of gene expression using curated sources from the literature. The second way to construct dynamic PPINs is based on expression variance [9] by determining the peak time points of expression for each protein. Thus, if a protein is at its peak point, it is considered to be in its active form, the status at which a protein can interact with its active neighbors. This assumption allows computing scored gene expression activity using a single threshold [10] or a systematical threshold [11]. In the present work, we assert that coexpression correlation may describe only the possible coregulated relationships of proteins, while existence of a specific interaction at a certain time point would depend on the activities of the two associated proteins. Hence, the integration of these two aspects becomes necessary in the construction of dynamic networks and a comprehensive way of defining the existence of dynamic PPIs is needed. In addition, some researchers argue that the gene expression data contain far more noise that will induce unauthentic factors. For example, the genes are sent into a filter that defines a criterion for genes of being dynamic or stable in Xiao et al.'s paper [12], and the stable ones are left out of the subsequent construction of dynamic networks. However, the definition of dynamic networks is slightly different from Xiao et al.'s. In our case, the stable active proteins are impartially included in the dynamic networks.

The second challenge of dynamic network analysis is to identify the most critical proteins out of a series of dynamic networks. As discussed above, Han et al. [7] concluded that hubs can be divided into two categories: the “party” and “date” hubs, among which the latter ones are more essential to global connectivity and functions that cells process. In this paper, the proteins exhibiting dramatic structural changes in the set of consecutive networks are defined as critical proteins which serve a compensation of the definition of “date” hubs to some extent. The intuition is that a series of networks in the same biological process should share a certain degree of consistence in structure. By extracting the consistence and reserving the structural difference of dynamic networks, we are able to find the critical proteins that are extremely important for the dynamic process. To this end, the consistent and varying properties of local structures in dynamic PPINs are studied in this work and a critical node detection method based on integration of multiple deep belief networks are proposed to identify the most critical proteins that are responsible for dynamic changes during a certain time period, and specifically, the case of yeast cell cycle processes is studied in our present work. There are several comparative methods of extracting consistent information from multiple graphs, such as the most straightforward average network and the joint nonnegative matrix factorization (JNMF) [13]. NMF tries to decompose the original graph to linear combination of basis vectors and is usually used in clustering problems, graph partition problems, and so on. In this work the hierarchical fashion of PPIN structures is taken into consideration by building a multisource integrated deep belief network (msiDBN) as a joint multilayer model that extracts the common higher levels of structural units. Our network is based on the previous work in [14]; however, in this msiDBN model, we decipher the structural varieties of nodes at different time points as the residuals of reconstruction and believe that the nodes with dramatic changes of structure in the networks play an important role in the progression of the cell cycle.

**Figure 1:**
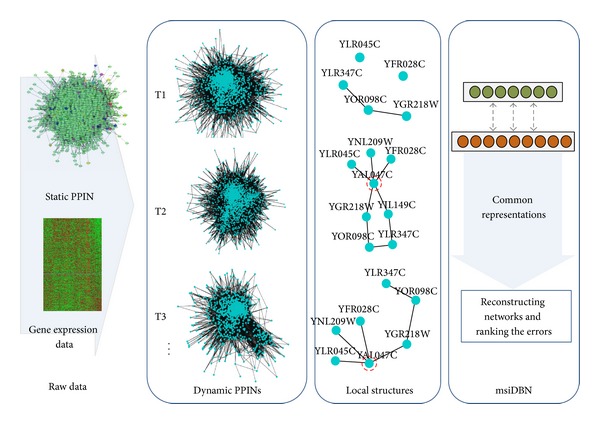
The framework of this paper.

The framework of this present work is shown in Figure 1. It is assumed that a small part of proteins in the network is associated with the changing of dynamic processes, marked by red circles in Figure 1 as an example. The changing of local structure is studied through our msiDBN method and in summary this work contributes in there ways.

- A new method for constructing dynamic coregulated PPINs has been proposed and it gets better representation of dynamic process by comparing to other construction methods.
- A multisource integrated deep belief network (msiDBN) is developed to extract the common representation of multiple networks, reconstruct the dynamic networks, analyze the residual of reconstruction, and identify the critical proteins in the yeast cell cycle processes.
- Experimental results show that our strategy of dynamic network construction is superior to the other baseline methods and the msiDBN is able to reconstruct the dynamic networks with the lowest root mean square error (RMSE) than the comparative methods for it extracts the consistent hierarchical structures while others do not have any deep insight of the networks.

The rest of the paper is organized as follows: the proposed dynamic PPIN construction method is described in Section 2; Section 3 defines the critical protein identification problem in an anomaly detection fashion and introduces the msiDBN method and the critical node ranking criteria; and the proposed methods are evaluated in Section 4 from different aspects. Finally, the conclusion of this work is given in Section 5.

## 2. Dynamic PPI Network Construction

In the traditional dynamic network construction methods, the instant interactions between proteins are determined using either coexpression correlation or gene expression level shift. However, we construct the dynamic networks by integrating both assumptions in the present work.

### 2.1. Activity Determination

Different peak time points of gene expressions may represent the dynamic changes in protein activities. Here we have assumed that proteins are active at their most peak points of gene expressions as discussed in Wang et al.'s work [11] and a similar threshold is set for the expression of each gene that is collected under continuous conditions. The active score is determined by

(1) $$\begin{array}{c} \text{AcScore}\left(p\right)=\text{th}{\text{r}}_{1}\left(p\right)\times F\left(p\right)+\text{th}{\text{r}}_{2}\times \left(1-F\left(p\right)\right), \end{array}$$

where thr_1_(*p*) is the mean of the gene expression of protein *p*, which is also denoted as *μ*(*p*), thr_2_(*p*) = *μ*(*p*) × *σ*(*p*), where *σ*(*p*) is the standard deviation of the gene expression of protein *p*, and *F*(*p*) = 1/(1 + *σ*(*p*)). As seen from (1), *F*(*p*) is a weight function of *σ*(*p*) and occurs in the range of (0, 1). An empirical parameter *α* was set for maintaining the active score AcScore within the range of (*μ*(*p*), *μ*(*p*) + *α*(*σ*(*p*)^3^/(1 + *σ*(*p*)^2^))). The performances of different empirical *α* have been discussed in the experimental section.

By setting such an active score threshold, the activity PPI networks Act_*t*_ were built for each timestamp:

(2) $$\begin{array}{c} \text{Ac}{\text{t}}_{t}=\delta_{t}\delta_{t}^{T}, \end{array}$$

where *δ*_*t*_ is a column vector representing the activity of proteins at time *t* and *δ*_*t*_^*T*^ is the transpose of the column vector. Each element in *δ*_*t*_ is determined by the binary threshold function as shown below:

(3) $$\begin{array}{c} \delta_{t}\left(p\right)=\{\begin{array}{cc} 1 & \text{if}\;g_{t}\left(p\right)\geq \text{AcScore}\left(p\right),\\ 0 & \text{if}\;\text{otherwise}. \end{array} \end{array}$$

### 2.2. Combining with Coexpression Correlation and Static PPIN

It has been demonstrated previously that functionally related genes are frequently coexpressed across multiple conditions and different organisms [15]. Coexpression correlation coefficient is used as a measure of coexpressed genes having the same expression variance patterns across different conditions [16]. We have used the Pearson correlation coefficient [17] (normalized to the range of 0 to 1) to calculate the coexpression correlation and build coregulation protein networks. Since the computation of correlation coefficient requires expression data that cover a period of time, a time window was set on the original expression dataset which covered the time points from *t* − 1 to *t* + 1, where t is the current time point. The correlation coefficient matrix at time *t* is denoted as CoE_*t*_. Combining the static PPIN and the activity PPIN provides the dynamic coregulation protein network at each time point:

(4) $$\begin{array}{c} A_{t}=\text{Co}{\text{E}}_{t}\circ \text{Ac}{\text{t}}_{t}\circ Ppi, \end{array}$$

where *Ppi* denotes the static PPI network adjacency matrix and ∘ represents element-wise multiplication.

Given the adjacency matrices of networks, the structural difference of networks can be studied in many different ways. The most important point is to incorporate the changes induced by neighbours' behaviors. Hence, we use higher order of the adjacency matrices to mimic random walks on these networks while keeping the nonnegative property at the same time.

## 3. Critical Node Detection Based on Multisource Integrated Deep Belief Network

### 3.1. Definition of Critical Node Detection Problem in Dynamic Networks

Given a set of PPINs {*A*_1_, *A*_2_,…, *A*_*T*_} under *T* time points, they are naturally evolving all along the biological process. The structure of network is represented by high order of adjacency matrix in this paper, which can be considered as the reachability of one node to the other in certain steps of rand walk. PPINs exhibit hierarchical structure and the trigger of changes in biological processes can be a small but complex set of molecules [18]. At a certain time point, the proteins in the PPIN are taken as nodes and each row of the high order adjacency matrix that a node corresponds to is seen as its feature at that time. There are *T* sources about the *N* nodes. To rank the most critical proteins, our intuition is that a node will receive low score if its topological structures of neighborhoods are consistent across the evolving networks and vice versa. It is impossible to directly compare the network structures because of the noise, sparsity, and indirect paths issues. However, because of the hierarchy of PPINs, we can extract hierarchical latent layers hidden in the networks that explain the evolution of network structures and protein functions. In other words, the hidden layers can be seen as the implied reasons of dynamic changes, by which the proteins fall into different groups of different characters of structural changes.

**Figure 2:**
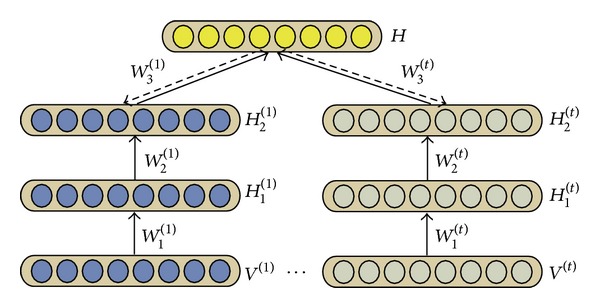
The flow of msiDBN.

The flow of msiDBN is shown in Figure 2 where *T* matrices of evolving networks are fed in as inputs. The msiDBN model tries to find the latent layers *H*_*l*_^(*t*)^, representing the hidden variables of the *l*th layer for the *t*th network and the symmetrical weighted connections between input layers and hidden layers, that is, *W*_*l*_^(*t*)^. As shown in Figure 2, multiple inputs are trained separately at the beginning and then are all combined to extract the common factors in the top layer.

### 3.2. DBN in Critical Protein Detection

**Figure 3:**
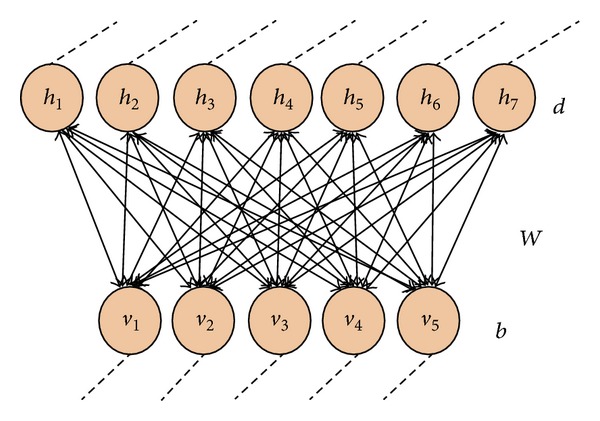
RBM in the DBN model.

To explain the framework of DBN, we should first go through the concept of restricted Boltzmann machines (RBMs), which are stacked one on top of each other to compose the DBNs [19]. RBM is defined as a network of symmetrically coupled binary random variables or units. As shown in Figure 3, these units can be divided into two groups: the visible variables, *v* ∈ {0,1}^|*v*|^, and the hidden variables, *h* ∈ {0,1}^|*h*|^ (|·| gets the dimension of the object inside it). The visible variables can be the original input or the transformed results from last layer according to the position of current RBM in the whole DBN model. The hidden variables imply the dependencies among the visible variables through their mutual interactional relationships as mimicked by the weighted matrix of *W*. In RBM, the interactions among visible-to-visible variables and among hidden-to-hidden ones are ignored [20]. Hence, we get a bipartite graph with completed connections.

The RBM defines an energy function between the visible and hidden layer variables:

(5) $$\begin{array}{c} E\left(v,h\right)=h^{T}Wv+d^{T}h+b^{T}v, \end{array}$$

where *h* and *v* are row vectors in *H* and *V*, respectively, *b* and *d* are the bias to the visible layer and hidden layer, and *W* is the weights between two layers. In RBM the training purpose is to learn the weights and biases between adjacent layers so that the energy function achieves its lowest level. The joint probability distribution of RBM with a normalization factor *Z* is

(6) $$\begin{array}{c} P\left(v,h\right)=\frac{1}{Z}E\left(v,h\right). \end{array}$$

With the restricted conditions, the hidden variables are independent given the visible variables and this property factorizes the individual activation probabilities of a hidden variable as follows:

(7) $$\begin{array}{c} P\left(h_{j}=1\;|\;v\right)=\text{sigmoid}\left(d_{j}+\underset{i}{\sum }W_{ij}v_{i}\right). \end{array}$$

Likewise, we have the individual activation probabilities of a visible variable as

(8) $$\begin{array}{c} P\left(v_{i}=1\;|\;h\right)=\text{sigmoid}\left(b_{i}+\underset{j}{\sum }W_{ij}h_{j}\right), \end{array}$$

where the sigmoid represents the logistic sigmoid function.

To train the probabilistic models, we typically adapt and find the best parameters that maximize the likelihood of the training data. The most straightforward way is to maximize the likelihood following the log-likelihood gradient. However, in the gradient of the log-likelihood, there are terms that are intractable, that is, the ones that compute the expectations over the joints of variables *v* and *h*. There are several ways of dealing with this problem, like the contrastive divergence (CD) [21] which uses a very short Gibbs chain to estimate the expectation of the joints of *v* and *h*. The reliability of CD has been proved by different groups of researchers [22–24].

The RBM model extracts the latent variables hidden in the training data and several RBMs are stacked one on top of others, using the hidden variables derived from lower models as the input, to get deeper layer variables that explain the hierarchical factorizations of PPINs. Given *l* layers of RBMs, the joint distribution is

(9) P(v,h1,h2,…,hl) =P(v ∣ h1)P(h1 ∣ h2)⋯P(hl−2 ∣ hl−1)P(hl−1 ∣ hl).

As the variables inside each layer are independent and considering the biases for each layer, we get

(10) $$\begin{array}{c} P\left(h_{li}=1\;|\;h_{l+1}\right)=\text{sigmoid}\left(d_{li}+\underset{j}{\sum }W_{lij}h_{(l+1)j}\right). \end{array}$$

### 3.3. Critical Protein Detection from Reconstruction of msiDBN

The msiDBN model to detect the critical proteins is built upon the assumption that most proteins have similar behavior patterns across the time courses while the most critical proteins that are responsible for the progression of the yeast cell cycle exhibit different expression levels and more importantly they engage in different interactions with contemporary neighbors. The other intuition is that the integrated deep belief networks extract the common features at the top layer (Figure 2) which represent the hidden deeper reasons for which the interactions change at different time points. Although we get merely *J* hidden variables at the top level, the feature space it can represent is scale of 2^*J*^ which is a much larger space than the common NMF clustering method gets. The joint probability of msiDBN is given as follows:

(11) $$\begin{array}{c} P\left(v^{\left(1\right)},\ldots ,v^{\left(t\right)},h\right)=P\left(h_{2}^{\left(1\right)},\ldots ,h_{2}^{\left(t\right)},h\right)\underset{t}{\prod }P\left(v^{\left(t\right)},h_{1}^{\left(t\right)},h_{2}^{\left(t\right)}\right), \end{array}$$

where *P*(*h*_2_^(1)^,…, *h*_2_^(*t*)^, *h*) is

(12) P(h2(1),…,h2(t),h)∝exp⁡(∑t∑idth2i(t)+∑kckhk   +∑t∑i,kh2i(t)W3(t)hk),

where *d*_*t*_ is the bias variable of *h* ^(*t*)^ and *c*_*k*_ is the bias of the top hidden variable *h*. As discussed above, the conditional distributions can be derived according to the independency conditions as follows:

(13) $$\begin{array}{c} P\left(h_{2}^{\left(t\right)}\;|\;h\right)=\text{sigmoid}\left(d_{t}+\underset{i}{\sum }h_{i}W_{3i}^{\left(t\right)}\right),\\ P\left(h\;|\;h_{2}^{\left(1\right)},\ldots ,h_{2}^{\left(t\right)}\right)=\text{sigmoid}\left(c+\underset{t}{\sum }\underset{i}{\sum }W_{3i}^{\left(t\right)}h_{2i}^{\left(t\right)}\right). \end{array}$$

The parameters of msiDBN can be learned approximately by greedy layer-wise training using CD. Therefore, with the common hidden variables and the trained weight matrices we are able to build an auto-encoder machine to reconstruct the input dynamic networks. The pseudo code in Algorithm 1 shows how to train the msiDBN model. With the common representation of multiple networks, we reconstruct them using (13) for the sampled data from *P*(*h*_2_^(*t*)^ | *h*) which can be viewed as the approximation of original data.

We quantify the reconstruction error using root mean square error (RMSE) which is denoted by Er and calculated as follows:

(14) $$\begin{array}{c} \text{E}{\text{r}}_{i}^{\left(t\right)}=\sqrt{\frac{1}{N}\overset{N}{\underset{j=1}{\sum }}{\left(A_{ij}^{\left(t\right)}-A_{ij}^{\left(R\right)}\right)}^{2}}, \end{array}$$

where *A*_*ij*_^(*R*)^ denotes the reconstructed network and Er_*i*_ ∈ ℝ^*T*^ is a vector representing the RMSE of protein *i* between the original data and reconstructed data across time 0 to *T*. The dispersion of Er_*i*_ is rated based on the relative standard error (RSD) which is RSD = *σ*/*μ*, with *μ* denoting the mean and *σ* denoting the standard deviation of Er_*i*_. The lower RSD scores correspond to the proteins that are well recovered by the model and also show average smoothness across time courses, while the higher RSD scores reveal the ones that are more likely having varying structures at different time points and are expected to play important roles during research like drug targets design.

## 4. Experiments and Results

### 4.1. Datasets

The gene expression data from GSE3431 [25] was used as the time course data to construct time course PPINs. GSE3431 is an expression profiling of yeast over three successive metabolic cycles. The overall design of this expression experiment is 12 time intervals per cycle, and approximately 25 minutes per time interval. Thus, for each gene there are 12 expression values at 12 time points in each cycle. In order to calculate the instant coexpression correlation coefficient, we choose *t* − 1, *t*, and *t* + 1 as three time points in a snapshot and at each time point there are three successive expression values serving as replicate samples. Particularly, for the first time point of the cell cycle, the last time point was chosen as its previous time point, and vice versa. Further, we also adopted another reference cell cycle gene expression data for yeast indexed by GSE7645 to alleviate the bias of expression in the calculation of mean and variance for each gene. In the experiment generating GSE7645,* S. cerevisiae* was cultured under oxidative stress induced by cumene hydroperoxide (CHP) and the transcriptional profile is collected at *t* = 0 (immediately before adding CHP) and at 3, 6, 12, 20, 40, 70, and 120 minutes after adding the oxidant.

The static PPIN of yeast was collected from BioGRID dataset for yeast and the cell cycle regulated protein dataset was downloaded from http://nar.oxfordjournals.org/content/38/suppl_1/D699 which will serve as the golden data in validation. We also constructed the cell cycle related static PPIN based on these proteins and their first neighbors in BioGRID PPIN. Finally we get a static PPIN with 2069 proteins and 43462 interactions between them.

The function of detected modules was validated by adopting the CYC2008 human-curated complex dataset as benchmark data [26]. CYC2008 is a comprehensive catalogue of manually curated 408 heteromeric protein complexes in* S. cerevisiae* reliably backed by small-scale experiments from the literature.

### 4.2. Evaluation of Dynamic Network Construction

We compare the proposed dynamic network construction method with traditional methods, that is, methods from the work of Tang et al. [10] and Wang et al. [11]. It is widely believed that the dynamic network reveals more accurate functional interactions between proteins than static PPIN and also a better dynamic network construction method should achieve better functional module analysis results. Hence, we run two traditional clustering methods on the different sets of dynamic networks and compare the precision of module detection results. Known complexes in the CYC2008 dataset served as a gold-standard data to evaluate the experimental results.

It is expected for a module detection method that the predicted clusters (*P*_*c*_) and the reference complexes (*R*_*c*_) match as much as possible. The overlapping scores OL(*P*_*c*_, *R*_*c*_) are used to find the matched complexes:

(15) $$\begin{array}{c} \text{OL}\left(P_{c},R_{c}\right)=\frac{\left|V_{P_{c}}\cap C_{R_{c}}\right|}{\left|V_{P_{c}}\right|\times \left|V_{R_{c}}\right|}, \end{array}$$

where |*V*_*P*_*c*__| is the size of the predicted cluster, |*V*_*R*_*c*__| is the size of the known complex, and |*V*_*P*_*c*__∩*V*_*R*_*c*__| is the number of the intersections of the predicted cluster and the known complex. *P*_*c*_ and *R*_*c*_ are considered to be matched if their OL score is larger than a threshold *σ*, which is typically chosen as 0.2 [27, 28]. Precision is defined as Prec = TP/(TP + FP), where TP (true positive) is the number of the predicted clusters matched with known complexes by OL > *σ*, and FP (False Positive) is the number of the unmatched known complexes in the predicted clusters.

#### 4.2.1. Comparison of Different Dynamic Networks Construction Methods

**Figure 4:**
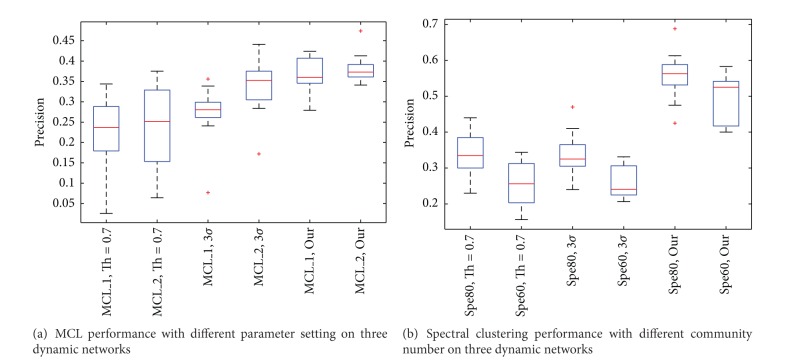
Different dynamic network construction methods.

In the experiments, a fixed threshold (Th = 0.7) was set to Tang's method, and the second construction method is described in Wang et al.'s paper [11] which uses a three-sigma threshold to determine the status of proteins.  Table 1 shows the numbers of active proteins derived from three different threshold setting methods. In our method, the parameter *α* was chosen to be 1.5. The performance of MCL and spectral clustering method on different dynamic PPI construction methods (Figure 4) indicates that our integrative method is more effective in constructing dynamic networks, and both functional module detection methods benefit from it by achieving the highest precisions compared to the other two dynamic PPIN construction methods.

#### 4.2.2. Parameter Setting

**Figure 5:**
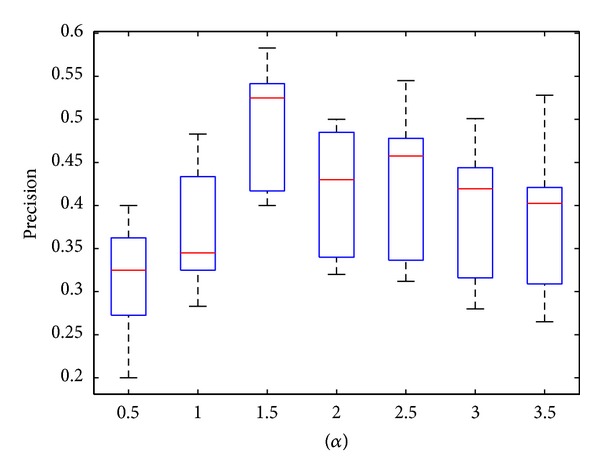
The distribution of precision under different parameter settings.

Initially, we analyzed the effect of change in parameters on our dynamic network construction method. Thus, we performed the spectral method to detect dynamic functional modules at 12 time points and compared the results with the CYC2008 dataset. The Precs of the results under different parameter settings have been compared as shown in Table 2 and the mean and variance of Prec are shown in Figure 5. From the results of comparison, it was obvious that on fixing *α* at 1.5 the precision of the functional module detection achieved the highest score. Thus, in the following comparisons with other module detection methods, this prime parameter setting has been used.

### 4.3. Critical Proteins Identification with msiDBN

In the msiDBN model, the visible input variables were chosen as the high order of the adjacency matrices; in this case the 2nd-order was used, and the self-transmissions were ignored which meant that the diagonal elements in each matrix were set to 0. We built the separate training DBNs as 2-layer models and combined different sources on the third layer as shown in Figure 2. We compared our method with two basic reconstruction methods and also with the original DBN to verify the effectiveness of our method.

An online resource of cell-cycle-related gene list from *Cyclebase* database was used as the golden data which contains 150 genes that are proved to be related to yeast cell cycle process. The evaluation metric of this experiment was also Prec with the same definition as the one introduced above.

**Figure 6:**
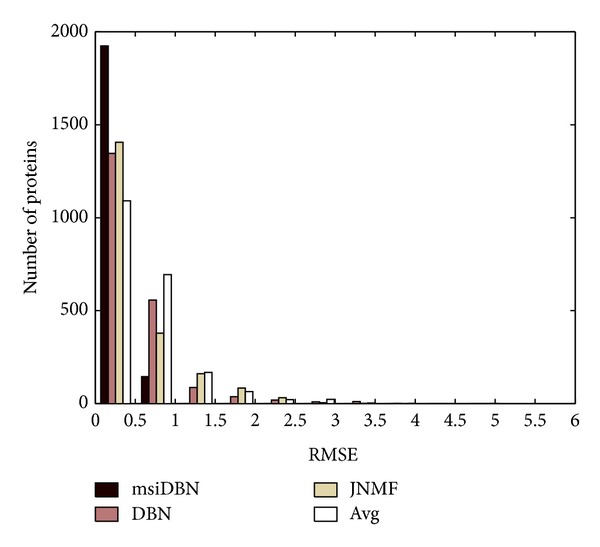
Comparison of RSME.

The baseline methods include joint NMF (JNMF) method, the straightforward average network, and the original DBN method. The JNMF method learns a common base matrix from different sources that best approximates the original sources. It is often used in clustering problems and dimension reduction problems. In our experiments the prior low dimension of JNMF was set as 500 by which the approximation to the original data generally achieved the best position. And the method which adopts the average network, denoted as AVG in the following content, simply extracted the average of the 2nd order adjacencies of the series of dynamic networks. Comparing with our msiDBN, the DBN method just processes our 12 networks through one straightforward deep structure of three layers to get the common representation and derive the reconstruction errors. By comparing the RMSEs, it is easy to see in Figure 6 that the msiDBN method obtains the best reconstruction while the AVG gets the worst in all of the four methods. The property of msiDBN is to extract a hierarchy of hidden features which naturally meets the characteristics of PPIN. The JNMF is analogous to one layer feature extraction model that does not fit in to its best within this scenario. In addition, our method surpasses the traditional DBN which considers all the networks identically and shows the promising results of the framework of multisource integrated deep belief networks.

**Figure 7:**
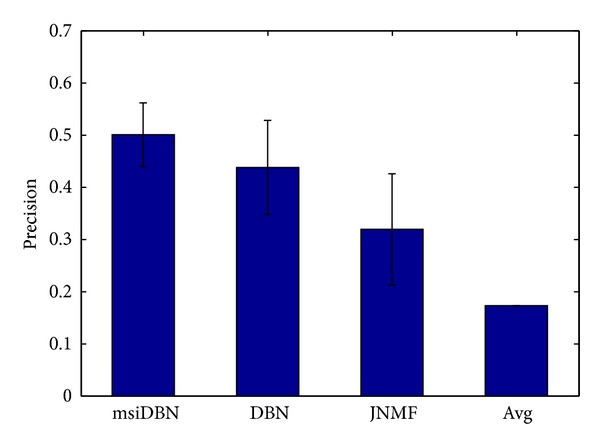
Precision comparison of different methods.

As we know, RSD is a measure that quantifies whether a set of variables are constant or have more variabilities. A high RSD number indicates that the data is more varied. The RSD scores of each protein were calculated and ranked to get the top 150 proteins in these three methods. The proteins in the top 150 RSD lists, derived from these three methods, were compared with the golden standard list from* Cyclebase* database. We ran msiDBN and JNMF 100 times separately to get the average performance and the comparison of precision results is shown in Figure 7. The matched proteins that are truly associated with cell cycle process in msiDBN were around 75, which was much higher than JNMF and AVG method. Among those unmatched proteins inside our top 150 list, we saw an interesting thing: according to the gene ontology annotations, a few of the unmatched proteins are relative to the true cell-cycle-related proteins, as shown in Table 3 where just a part of the unmatched proteins are listed due to limitations on space. We also checked in the static PPI network and discovered that most of the unmatched proteins are directly linked to the cell-cycle-related proteins or within short length of link distance. This phenomenon is consistent with our structural changing assumption about critical proteins in which for a critical protein, which has been varied structure during the cell cycle progression, the structure of its neighbors must change along with it.

## 5. Conclusion

In this paper, the structural variability of dynamic PPINs was studied to identify the critical proteins in the yeast cell cycle process. A comprehensive method of constructing dynamic active PPI networks was proposed which simultaneously modeled the activity of proteins and assembled the dynamic coregulation protein network at each time point. And then a critical node detection method that integrated multiple networks into deep belief network model was developed, in which the reconstruction results were ranked by the variabilities of the reconstruction errors across time courses and finally the top proteins in rank order were selected to be the critical ones that may play important roles in dynamic mechanisms. We evaluated our network construction method by comparing the functional representations of proteins in the derived networks with that from two traditional construction methods and our method achieved superior function analysis results. The critical protein ranking results from msiDBN were compared with results from JNMF reconstruction method and the comparison of results showed that msiDBN had better reconstruction rate and identified more proteins of critical value to yeast cell cycle process.

The fact that a few proteins among the unmatched protein lists are truly relevant to the cell cycle process inspires an interesting idea that the system analysis of dynamic networks should be done to reveal groups of critical proteins with the same or relative functional roles in the dynamic mechanism. We will focus more on a system level study of the dynamic networks in the future research.

## Figures and Tables

**Algorithm 1:**
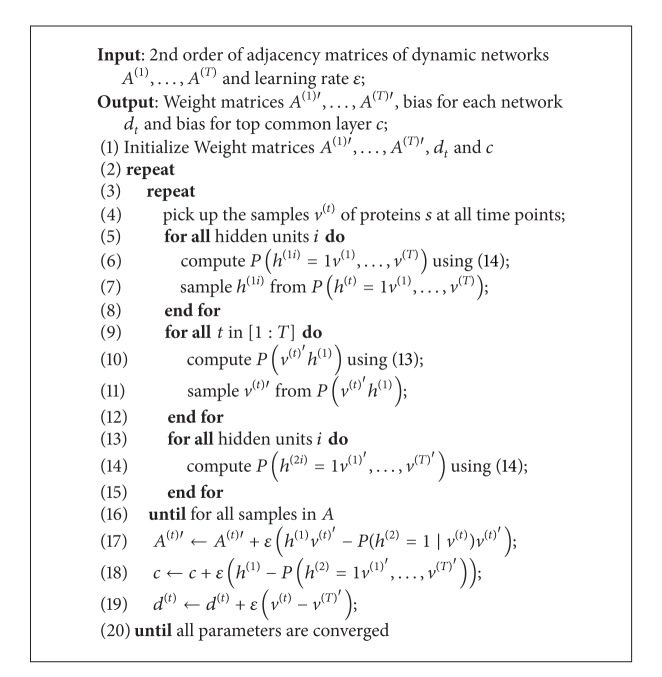
Multisource integrated deep belief nets (msiDBNs).

**Table 1:** Active proteins and their interactions in different dynamic networks.

Methods	*T*1	*T*2	*T*3	*T*4	*T*5	*T*6	*T*7	*T*8	*T*9	*T*10	*T*11	*T*12
**Our, AP**	1068	1010	1019	882	853	828	898	1057	1195	1031	1086	1036
**Our, Ins**	10489	9890	10733	8100	6975	6609	7662	11584	15906	11850	12857	11040
Th = 0.7, AP	1071	1080	1062	962	902	842	843	1066	1162	991	1051	885
Th = 0.7, Ins	12267	13034	13653	10463	9345	8370	8133	13263	16302	13586	13640	10010
3segma, AP	531	545	505	393	364	343	361	603	688	473	545	449
3segma Ins	3003	3325	3140	1841	1462	1278	1588	4352	6380	3654	4123	2611

**Table 2:** Parameter settings, *α*∈(0.5~3).

*α*	*T*1	*T*2	*T*3	*T*4	*T*5	*T*6	*T*7	*T*8	*T*9	*T*10	*T*11	*T*12	Average
0.5	0.270	0.350	0.325	0.325	0.375	0.350	0.200	0.375	0.225	0.400	0.275	0.325	0.316
1	0.340	0.483	0.483	0.317	0.350	0.417	0.283	0.450	0.400	0.333	0.333	0.317	0.376
**1.5 **	**0.533**	**0.517**	**0.483**	**0.400**	**0.417**	**0.417**	**0.400**	**0.550**	**0.583**	**0.533**	**0.567**	**0.533**	**0.494**
2	0.330	0.400	0.480	0.350	0.320	0.320	0.380	0.460	0.490	0.490	0.460	0.500	0.415
2.5	0.470	0.387	0.445	0.328	0.345	0.312	0.320	0.478	0.495	0.545	0.478	0.470	0.423
3	0.423	0.394	0.437	0.280	0.287	0.316	0.316	0.451	0.473	0.501	0.423	0.416	0.393
3.5	0.390	0.371	0.421	0.315	0.290	0.265	0.303	0.421	0.528	0.453	0.415	0.415	0.382

**Table 3:** GO enrichment of unmatched proteins in the top 150 list.

Protein	GO-ID	Term description
YGL016W	GO:0016021	Integral to membrane
	GO:0006606	Protein import into nucleus

YKL203C	GO:0000080	G1 phase of mitotic cell cycle
	GO:0007049	Cell cycle
	GO:0030037	Actin filament reorganization involved in cell cycle

YBR078W	GO:0031505	Fungal-type cell wall organization
	GO:0031225	anchored to membrane
	GO:0005618	cell wall

YLL031C	GO:0015867	ATP transport
	GO:0009277	fungal-type cell wall

YBR122C	GO:0032543	Mitochondrial translation
	GO:0005762	mitochondrial Large ribosomal subunit
	GO:0005739	Mitochondrion
